# The Evaluation of the Suitability, Quality, and Readability of Publicly Available Online Resources for the Self-Management of Fear of Cancer Recurrence

**DOI:** 10.3390/curroncol31010005

**Published:** 2023-12-22

**Authors:** Verena Shuwen Wu, Tiyasha Sabud, Allan ‘Ben’ Smith, Sylvie D. Lambert, Joseph Descallar, Sophie Lebel, Adeola Bamgboje-Ayodele

**Affiliations:** 1 Faculty of Medicine and Health, South West Sydney Clinical Campuses, University of New South Wales (UNSW Sydney), Liverpool, NSW 2170, Australia; 2 Ingham Institute for Applied Medical Research, Liverpool, NSW 2170, Australia; 3 The Daffodil Centre, The University of Sydney, A Joint Venture with Cancer Council NSW, Sydney, NSW 2006, Australia; 4 Ingram School of Nursing, McGill University, Montreal, QC H3A 2M7, Canada; sylvie.lambert@mcgill.ca; 5 St. Mary’s Research Centre, Montreal, QC H3T 1M5, Canada; 6 School of Psychology, University of Ottawa, Ottawa, ON K1N 6N5, Canada; 7 Biomedical Informatics and Digital Health, School of Medical Sciences, Faculty of Medicine and Health, The University of Sydney, Sydney, NSW 2006, Australia

**Keywords:** fear of cancer recurrence, self-management, cancer, oncology, health resources, internet, readability, suitability, quality, health literacy

## Abstract

Cancer survivors often rely on the internet for health information, which has varying levels of readability, suitability, and quality. There is a need for high-quality online self-management resources for cancer survivors with fear of cancer recurrence (FCR). This study evaluated the readability, suitability, and quality of publicly available online FCR self-management resources. A Google search using FCR-related keywords identified freely available FCR self-management resources for cancer survivors in English. Resource readability (reading grade level), suitability, and quality were evaluated using relevant assessment tools. Descriptive statistics and cluster analysis identified resources with higher suitability and quality scores. Mean resource (*n* = 23) readability score was grade 11 (SD = 1.6, Range = 9–14). The mean suitability score was 56.0% (SD = 11.4%, Range = 31.0–76.3%), indicating average suitability and the mean quality score was 53% (SD = 11.7%, Range = 27–80%), indicating fair quality. A cluster of 15 (65%) resources with higher suitability and quality scores was identified. There were no significant associations between suitability or quality scores and the type of organisation that published the resources. Online FCR self-management resources varied in readability, suitability and quality. Resources with higher quality and suitability scores relative to other resources are identified for use by healthcare professionals and cancer survivors. Resources that are more culturally appropriate, with lower reading grade levels and detailed self-management strategies are needed.

## 1. Introduction

Fear of cancer recurrence (FCR), defined as “the fear, worry, or concern about cancer returning or progressing” [1], is a prevalent issue among cancer survivors with more than half reporting moderate FCR and 19–45% experiencing high/clinical FCR [2]. Additionally, FCR is frequently identified by survivors as a major concern and one of their most common unmet supportive care needs across multiple cancer sites [3,4]. FCR manifests on a continuum, ranging from rational concerns about recurrence to clinically significant FCR, characterised by persistently high levels of worry/preoccupation and hypervigilance to bodily symptoms [1,5]. FCR can be present from diagnosis and remain stable or worsen throughout survivorship, particularly when these fears go unaddressed [6,7]. High levels of FCR are related to poorer mental health and quality of life in both cancer survivors [4] and caregivers [8] and greater healthcare use [9], burdening both cancer survivors, their caregivers and the healthcare system.

Stepped or matched care models, where recommended interventions are matched to FCR level reported have been proposed and piloted as a means of efficiently and effectively addressing cancer survivors varying levels of FCR [10]. Delivery of matched care requires interventions of varying intensity, from self-management resources that can be provided to all cancer survivors, or those with minimal/mild FCR, as part of an initial step/universal care, through to therapist-delivered interventions for more severe FCR. Provision of less intensive interventions early in survivorship may mitigate worsening of FCR over time [11]. 

To date, largely face-to-face psychologist-delivered treatments targeting more severe clinical levels of FCR have demonstrated efficacy in reducing FCR and ameliorating associated psychological distress and quality of life impairments. A meta-analysis of 23 controlled trials [7] evaluating the effect of psychological interventions on FCR, 20 of which were delivered face-to-face, found post-treatment improvements in FCR that were maintained at follow-up. While these interventions may offer an effective means of addressing more severe FCR, barriers, including time and costs associated with travel and taking time off work for treatment, may hinder accessibility [12,13,14]. Limited research has focused on interventions that can feasibly address moderate but burdensome levels of FCR. Brief interventions delivered by non-mental health professionals (e.g., nurses or oncologists) have demonstrated potential [15,16,17], but require significant investment in education and training to deliver [18]. 

Self-management, defined as the active management of one’s own physical and psychosocial wellbeing, may help overcome the barriers of face-to-face interventions described above [19]. Low-intensity interventions, such as online self-management resources that provide psychoeducation about FCR, and strategies for managing FCR, may help make FCR support more widely available to survivors experiencing minimal or moderate FCR. Online psychological interventions for FCR have demonstrated moderate, but variable, effects in reducing FCR [20]. While these online interventions may increase access to effective FCR treatment, many still require a substantial time investment (e.g., about 5 h for iConquerFear [14]), which may make them unappealing for some cancer survivors with mild-moderate FCR. Further, FCR reductions over time in the attention control group in the trial of another online intervention (FoRtitude) suggest that for some survivors with moderate FCR, information alone may be sufficient to reduce FCR, particularly those with higher self-efficacy, which was found to predict FCR improvements [21]. 

Approximately 69% of cancer survivors rely on the internet for health information [22]. There are large amounts of cancer-related information available online with varying levels of readability, suitability, and quality [23,24]. The lack of consistent guidelines and regulation of online health information risks inaccurate or low-quality information being unhelpful or even harmful [23]. Unmet information needs and inadequate information provision have been theorised [25] and found to be related to greater FCR [16]. A recent review of 45 online resources for adult cancer survivors who have completed primary treatment was conducted and their quality, suitability, readability, and usefulness in addressing prevalent unmet needs were assessed [26]. Findings showed that only 58% of these unmet needs were addressed, for which cancer recurrence was only partially addressed (mean = 0.6/1.0) [26]. There is a clear need for high quality low-intensity online self-management resources for cancer survivors with mild to moderate FCR that are suitable and readable. These resources may prevent FCR from worsening and reduce the impact on mental health and quality of life. To fill this gap, we aimed to evaluate the suitability, quality, and readability of publicly available online resources designed to enable cancer survivors with mild-moderate FCR levels self-manage their concerns.

## 2. Materials and Methods

### 2.1. Sample and Setting

Online resources qualified as a self-management resource if they included suggestions for cancer survivors about how to manage FCR, as opposed to mere information provision about the FCR experience. To evaluate online self-management resources for FCR, an online search replicating how cancer survivors might search for FCR information online was conducted. Consumers commonly locate health information by entering short phrases and engaging with websites returned on the first page [24]. As such, combinations of common keywords and layperson phrases were formulated after consultation with the librarian at the Liverpool Hospital (South West Sydney Clinical Campuses). The autocomplete function on Google Search also informed the development of search terms as the suggested phrases are generated based on queries commonly entered in the search engine. The author BS reviewed and approved the search terms (see Table 1). 

The search terms were then entered into Google Search sequentially using the Google Chrome web browser, connected to an Australian IP address. Browsing history, cookies, and cache files were deleted prior to searching to ensure that the retrieval of Uniform Resource Locators (URLs) was not affected by search history and preferences. The search was conducted in Sydney, Australia (25 February 2020). The first page of results for each keyword or phrase were collated in a Google Document by TS with the title and URL of the originating resource noted and screened against the eligibility criteria by TS and VW. 

Resources were included if they were freely available online (e.g., webpages, videos, or downloadable fact sheets) in English; addressed cancer survivors directly; and provided detailed information on FCR and management options. Questionnaires, testimonials, scientific journal articles, advertisements, books, podcasts, and resources targeting healthcare professionals were excluded. Resources eligible for inclusion were entered in a Microsoft Excel spreadsheet which was used for the subsequent evaluation of their characteristics, readability, suitability, and quality.

### 2.2. Data Collection

Resources were evaluated for readability, suitability, quality, and other characteristics (e.g., location, type). Readability is the ease with which sentences and paragraphs can be understood by the reader [27]. Suitability is the level of comprehension and acceptability of written information [28]. Quality refers to the integration of evidence-based self-management advice [29,30]. Ten (43.5%) resources were independently rated by TS and VW, with ratings compared to establish inter-rater reliability (see details Section 2.2.5). Rating discrepancies were resolved through discussion with BS. Remaining resources were assessed by TS.

#### 2.2.1. Readability Indices

Readability was assessed using the Gunning Fog Index (GFI) [31], the Simplified Measure of Gobbledygook (SMOG) [32], and the Flesch-Kincaid Grade level [33] using an online tool [34]. GFI [31] and SMOG [32] estimate years of formal education required to understand the text on first reading. The Flesch–Kincaid Grade Level indicates the difficulty of a passage and the required reading grade, as per school grade levels in the United States (US) [33]. Lower levels (ideally 8th grade and below) correspond to higher readability. All raw text from each resource was compiled in a Google Document [35], and manually formatted so that images, tables, and irrelevant text (e.g., hyperlinks and advertisements) were removed. The formatted text was then pasted into the readability tool [34]. To account for variability in scores generated by the different measures, a mean reading grade across these three indices was calculated [36,37]. 

#### 2.2.2. Suitability

Suitability was assessed using the Suitability Assessment of Materials (SAM) [28], a validated 22-item assessment tool [38,39]. SAM appraises six categories affecting readability and comprehension [28]: content, literacy demand, graphics, layout and typography, learning stimulation/motivation, and cultural appropriateness. Each category was assessed using 2–5 items rated from 0 (not suitable) to 2 (most suitable), with higher scores representing greater suitability. Item scores for each category were summed, divided by the total possible score per category, then converted to a percentage, whereby superior = 70–100%, adequate = 40–69%, and unsuitable < 39% [28].

#### 2.2.3. Quality 

Quality was assessed using DISCERN [29], a validated 16-item grading tool [23,40] that appraises the quality of the material relating to publication reliability (8 items), quality of suggested treatment (i.e., self-management) options provided (7 items) and overall quality rating (1 item). Each item was rated from 1 to 5, with higher scores reflecting better quality. Scores on items 1–15 were summed, and a percentage was calculated based on a total possible score of 75, where excellent = 84–100%, good = 68–83%, fair = 52–67%, poor = 36–51%, and very poor < 35% [29].

#### 2.2.4. Resource Characteristics 

Additional information about the resources, including location, responsible organisation name, organisation category (i.e., cancer support organisation, medical/research centre, professional association, health education/publishing), format (e.g., website versus online booklet/PDFs), year updated, audio-visual elements (yes/no), presence of self-management skills (yes/no) and advertisements (yes/no) were extracted to determine their association with resource quality and suitability.

#### 2.2.5. Bias: Inter-Rater Reliability 

Intraclass Correlation Coefficient (ICC) estimates and their 95% confidence intervals were calculated on the SAM and DISCERN total scores using IBM SPSS Statistics 26 (IBM Corp., Armonk, NY, USA), based on a mean rating (k = 2), consistency, two-way random-effects model [41]. 

### 2.3. Data Analysis

Descriptive statistics were calculated to summarise the overall readability, suitability and quality of the resources. To identify groups of resources scoring high versus low for both quality and suitability, a hierarchical cluster analysis using Ward’s linkage [42,43] was performed on SAM and DISCERN scores. Characteristics of resources in each cluster were compared using Fisher’s Exact Test (*p* < 0.05). Characteristics of resource associated with high SAM or DISCERN scores (not both as in the cluster analysis) were also assessed separately using *t*-tests [44]. All the analyses were performed using IBM SPSS Statistics 26 (IBM Corp., Armonk, NY, USA).

## 3. Results

### 3.1. Search Results 

Thirty-seven online resources were identified and screened for eligibility. Fourteen were subsequently excluded for the following reasons: (1) inadequate information regarding FCR self-management (i.e., minimal or no description about how to manage FCR) (*n* = 8); (2) redundancy (multiple retrievals from Google using different search terms; *n* = 4); (3) not targeted towards cancer survivors (*n* = 2). Twenty-three resources were included in the final sample (see Table 2). 

### 3.2. Resource Characteristics

Of the included resources, three quarters (*n* = 17, 74%) were from the US. More than half (*n* = 15, 65.2%) were published by national cancer organisations and less than a quarter (*n* = 4, 17%) by independent health organisations (e.g., Mayo Clinic Health System [45]). 78% (*n* = 18) of resources were webpages as opposed to downloadable fact sheets or booklets, only two (9%) resources contained videos. Nine (39%) resources contained advertisements. See Appendix A for additional resource characteristics.

### 3.3. Inter-Rater Reliability

An ICC of 0.511 indicated moderate inter-rater reliability for suitability and quality scores where <0.5 = poor, 0.5–0.7 = moderate reliability, 0.75–0.9 = good, and >0.9 = excellent [41].

### 3.4. Readability 

Readability was assessed by calculating the average of the GFI, SMOG, and Flesch–Kincaid Reading Grade Level, with higher reading grade levels indicating lower readability. Average reading grade scores ranged from grade level 9–14, which can be interpreted as ranging between secondary to tertiary school level. The mean reading grade score was grade level 11 (SD = 1.6), equivalent to secondary school. 

### 3.5. Suitability 

The mean SAM score was 56.0% (SD = 11.4%, Range = 31.0–76.3%) indicating ‘adequate’ suitability. Only one resource (4.3%) was rated ‘superior’ (Cancer survivors: Managing your emotions after cancer treatment by Mayo Clinic Health System [46]); two (8.7%) were ‘not suitable’. The remaining 87.0% (*n* = 20) were in the adequate range (see Table 3). Content (mean = 1.34/2, SD = 0.80) and literacy demand (mean = 1.33/2, SD = 0.76) were rated highest, whereas graphics (mean = 0.67/2, SD = 0.64) and cultural appropriateness (mean = 0.95/2, SD = 0.20) were rated lowest.

### 3.6. Quality 

The average DISCERN score for the entire sample was 53% (SD = 11.7; range = 27–80%). 15/23 resources (65.2%) scored ≥50%. Only one resource [47], scored 5/5 on the overall quality item. The mean score for the reliability category (2.92/5) was higher than the mean score for the treatment choices category (2.32/5) (see Table 4). The highest rated DISCERN items related to clarity of aims (Mean = 4.3, SD = 1.06), relevance (mean = 4, SD = 0.8), and achievement of aims (Mean = 3.86, SD = 0.96). The lowest rated items related to treatment (i.e., self-management) risks (mean = 1.04, SD = 0.2), outcomes without treatment (mean = 1.21, SD = 0.42) and source of information (mean = 1.74, SD = 1.48).

### 3.7. Cluster Analysis (Suitability and Quality)

There was a moderately positive correlation (r = 0.77) between SAM and DISCERN percentage scores. Hierarchical cluster analysis of SAM and DISCERN scores indicated two groups: Cluster 1 contained 15 resources with higher scores for both SAM (mean = 62.57, SD = 5.72) and DISCERN (mean = 59.13, SD = 7.9), while Cluster 2 contained 8 resources with lower scores for SAM (mean = 43.48, SD = 8.33) and DISCERN (mean = 41.91, SD = 9.19). There was a significant increase in the mean SAM and DISCERN scores in cluster 1 compared with cluster 2 (SAM t(21) = 6.5, *p* < 0.001, DISCERN t(21) = 4.71, *p* < 0.001). 

### 3.8. Characteristics of Resources with Higher Suitability and Quality

There were no significant differences between organisation category and cluster (Fisher exact test, *p* = 0.318). Resources published in Australia (*n* = 4/4, 100%) were more likely to be included in the superior resource cluster compared with USA (*n* = 5/17, 29%) (Fisher exact test, *p* = 0.0211). Being published in the UK or Australia was independently associated with significantly higher mean quality (DISCERN t(21) = 2.1, *p* = 0.048) and suitability (SAM score t(18.08) = 3.13, *p* = 0.006). No significant associations were found for the other characteristics (format, year updated, audio-visual elements, presence of self-management skills, and advertisements).

## 4. Discussion

This is the first study to evaluate the suitability, quality, and readability of publicly available online resources for the self-management of FCR, despite FCR being a highly prevalent concern among cancer survivors. Twenty-three resources were evaluated, 15 of which were of higher suitability and quality (Cluster 1), and 8 were of lower suitability and quality (Cluster 2). The type of organisation did not impact on the suitability and quality of the resources. However, resources with higher suitability and quality were more likely to be published in the UK or Australia. None of the resources met the criteria for acceptable readability (8th grade and below).

The internet is a common source of cancer-related information. Despite this, our findings indicated low readability of online resources for FCR self-management. Readability of all resources was higher than the recommended 8th grade level [48]. The high reading grade of the included resources, indicating more difficult text requiring higher levels of education to understand, complements a recent evaluation of publicly available resources for adult cancer survivors who have completed treatment, where the average reading level was 11 (equivalent to secondary school) with only one resource meeting the recommended 8th grade [26]. These findings are also consistent with an evaluation of online resources for self-management of depression, which found that readability of the included resources required at least secondary/high school level education [30]. Another study which evaluated the readability of Australian online health information (covering 12 common health conditions including cancer) found 251 web pages from 137 websites had an average reading grade of 10.5 (Flesch–Kincaid)/12.1 (SMOG), considerably higher than the average Australian reading level of grade 8 [48]. Low readability of online resources increases the likelihood of individuals disregarding or misinterpreting health information, potentially leading to self-management deficiencies and worsening of health outcomes [48,49]. This risk is amplified for cancer survivors of differing ethnic/cultural backgrounds, with limited fluency in the dominant language of the country they reside in, and/or who have low health literacy. Readability of online FCR self-management resources needs to be improved for these resources to benefit the broad spectrum of cancer survivors affected by FCR. 

Only one resource was rated ‘superior’ in terms of suitability, while the rest were rated as ‘adequate’. There was limited use of graphics, learning, stimulation, and motivation strategies (e.g., interactive features, behaviours modelled/specific, and motivation/self-efficacy) across resources. Similarly, limited use of graphics were found for online resources for cancer survivors, which the authors note may further compound readability issues [26]. This is problematic, as these strategies are likely to enhance learning, engagement and retention of skills, which are critical to enabling self-management, [38]. The resources also demonstrated limited cultural appropriateness, with low ratings regarding congruency of presented information with the logic, language, and experiences of cultural backgrounds other than the White majority. This result may be partly because we only included English-language resources in this study, which typically did not target a specific cultural group, and SAM, as a literacy assessment tool, has been criticised for its limited application when there is limited information about the intended cultural audience [36]. However, it is essential that FCR self-management resources cater for people from culturally and linguistically diverse backgrounds considering the growing diversity of the many countries populations and the fact that FCR can impact Indigenous and minority people differently [50]. 

The quality of the evaluated resources was generally moderate, although this did not significantly differ across categories. One of the common issues diminishing quality was a limited reporting of information sources for resource content, for this also presents as a problem for online resources targeted towards cancer survivors who have completed treatment [26]. Transparent attribution of information sources is a key element impacting judgements about credibility, as this enables readers to verify the information independently [51]. This is a common issue with online health information generally [52,53]. In this instance, it may impact on engagement with online FCR self-management resources, and if information is not evidence-based, could also lead to misleading or unhelpful recommendations.

Included resources rated slightly worse in terms of outlining FCR self-management options than presenting reliable information. Many resources failed to describe benefits and risks associated with each recommended approach to self-managing FCR, and what would happen without self-management/treatment. Similarly in the evaluation of online resources for cancer survivors who have completed treatment, there was minimal description of the risks associated with the suggested self-management strategies [26]. Presenting information about risks and benefits of FCR self-management strategies is critical to informed decision-making, as sometimes self-management strategies may not benefit survivors [11] and in some cases focusing more on FCR may increase distress, especially in people who typically use avoidant coping. Describing potential outcomes of foregoing FCR self-management may motivate survivors to try self-management, considering that evidence suggests that for many cancer survivors FCR often persists and sometimes worsens if left unmanaged [4,54]. 

This was the first study to evaluate publicly available online self-management resources for FCR. Study strengths include use of search methodology likely to reflect common searches for online information about FCR by cancer survivors and validated tools for assessing readability, suitability, and quality. Study limitations include only resources published in English being evaluated, meaning our findings are only relevant to patients literate in English. The search for resources was conducted in 2020, which means that there may be more recently developed or updated resources that have been missed. Nonetheless, this study provides a useful framework for developing and evaluating FCR self-management resources and identifies resources of high quality and suitability for use in practice. Future research could extend our work by adopting the same approach in evaluating the suitability and quality of the FCR self-management resources published more recently and in other languages, seeing as FCR seems to affect cancer survivors regardless of where they live [2]. This is particularly important, as immigrant patients, who have been found to have greater unmet needs regarding FCR, [50,55] may turn to information published in their language from their home country, as they commonly do for other forms of health information [56]. While we used validated instruments to assess resource readability, suitability and quality, the DISCERN instrument used to assess quality was originally designed to assess the quality of information regarding medical treatments rather than self-management strategies, which complicated assessment of resource quality. 

While we assessed resource accessibility in terms of their readability and suitability (e.g., content, layout, and typography), we did not evaluate more technical factors impacting accessibility (e.g., user interface and web functionality that contribute to ease of navigation, readability, and comprehension) [57]. Future development and evaluation of online FCR self-management resources could use the Web Content Accessibility Guidelines (WCAG), which outline technical requirements and features that enable easy and effective website use [58]. Although these guidelines were developed to improve web accessibility for people with disabilities, they can also be applied to ensure usability for other populations, such as people affected by cancer. Finally, there is a need for data regarding which resources are commonly recommended by oncology health professionals, used by cancer survivors and their effects on FCR to enable correlation of resource readability, suitability and quality with usage and impact. 

### Practical Implications 

This review identifies some key areas for improvement in the readability, suitability and quality of existing online FCR self-management resources and factors for future resource developers to consider. Readability can be enhanced by using readability tools (such as the online tool used in our review) to evaluate resource content and ensure the language used fits the literacy level of the general population. Resource suitability can be improved through greater use of graphics to aid reader comprehension, incorporating learning stimulation and motivation (e.g., posing scenarios or questions for readers) to promote long-term retention of new knowledge/skills, and modelling specific self-management behaviours (e.g., instructions presented in steps or video format) to facilitate learning. Quality can be enhanced through transparent reporting of information sources (e.g., providing links to the original sources), balanced description of the benefits and risks for each self-management strategy, and clear description of potential consequences if a condition, such as FCR, is left unmanaged. Development of future resources could apply the readability, suitability (SAM) and quality (DISCERN) tools used in our evaluation to avoid identified resource shortcomings. Involving consumers in the development of resources may also help ensure that they present content that suits the needs and preferences of the target population [59].

For patients and clinicians seeking additional FCR self-management support resources, our results provide a useful guide for which resources to consider (see Table 2 for resources ranked by quality rating). Provided they can understand the resource contents, many patients and clinicians may not discern/have a preference where a resource comes from, but some may prefer local resources. It is worth noting that Australian and UK resources were generally of higher suitability and quality, while the suitability and quality of resources from the USA were more variable, so those based in the USA may wish to be more selective. High quality and suitability FCR self-management resources could also serve as a form of ‘universal care’ for FCR provided to all survivors as part of a stepped care approach to managing FCR, which could help prevent FCR from worsening [10]. There is preliminary evidence from the Fear-Less study suggesting that stepped care models including an FCR self-management resource as the initial step can reduce FCR [11]. However, it should be noted that the FCR self-management resource in the Fear-Less study was more intensive and included some therapist guidance, compared with the typically brief and entirely self-management resources evaluated here.

## 5. Conclusions

Many cancer survivors rely on the internet for self-management information and are affected by FCR. Consequently, it is essential that publicly available online FCR self-management resources deliver high quality, evidence-based information that can be read and understood by the diverse spectrum of people living with and beyond cancer. There is potential for improvement of the readability, suitability and quality of existing FCR self-management resources, particularly in terms of readability, which required a much higher level of education than recommended. As well as improving existing resources, further research is needed to evaluate FCR self-management resources in languages other than English, and from non-Western countries.

## Figures and Tables

**Table 1:** Search Terms.

Search Terms
How to manage fear of cancer recurrence Anxiety and cancer Cancer patients and fear of cancer recurrence Manage fear of cancer recurrence How to stop worrying about fear of cancer recurrence How to fight emotions regarding cancer recurrence How to deal with fear of cancer recurrence What if my cancer comes back? Fear of cancer recurrence—Self manage Fear of cancer recurrence self-help cancer institute Fear of cancer recurrence Self care Online management of Fear of Cancer coming back Fear of cancer recurrence—Self management How to self-manage worries about cancer coming back Fear of cancer recurrence—Self help Tools to manage fear of cancer recurrence How to beat cancer phobia Cancer and anxiety management Fear of cancer recurrence and ways to manage it How can I manage my anxiety about my cancer returning Coping with fears of cancer coming back Fear of cancer recurrence and American cancer society Conquering cancer recurrence fears How to stop thinking about my cancer coming back How to stop thinking about cancer recurrence Ways to manage fear of cancer recurrence Ways to manage fear of cancer coming back How to help myself with fear of cancer recurrence Coping with fear of cancer recurrence Fear of recurrence and cancer Coping with cancer Fearful of cancer returning Tips and strategies to cope with fear of cancer recurrence Prevent worries about cancer coming back Online coping strategies for fear of cancer recurrence Fear of cancer recurrence patient education How do I stop worrying the cancer will come back

**Table 2:** Characteristics of included FCR self-management resources.

Cluster *	Title and Link	Year Updated	Quality (DISCERN) Rating (%)	Suitability (SAM) Rating (%)	Mean Reading Grade Level	Resource Format	Resource Location	Responsible Organisation (Organisation Category)
1	Coping with the fear of cancer coming back (fear of cancer recurrence) https://www.petermac.org/sites/default/files/media-uploads/ACSC_Factsheet_FearOfCancerComingBack.pdf (accessed on 25 February 2020)	2017	Good (80%)	Adequate (61.9%)	10	PDF/e-book	Australia	Peter MacCallum Cancer Centre (Medical/research center)
1	How to deal with FCR—patient treatment and support https://www.fredhutch.org/content/dam/www/research/patient-treatment-and-support/survivorship-program/survivorship-health-links/Fear%20of%20Recurrence.pdf (accessed on 25 February 2020)	2011	Fair (66.7%)	Adequate (65.6%)	11	PDF/e-book	USA	Fred Hutchinson Cancer Research Centre (Medical/research center)
1	Fear of cancer returning https://www.maggies.org/cancer-support/managing-emotions/fear-cancer-returning/ (accessed on 25 February 2020)	2020	Fair (65.3%)	Adequate (65.8%)	11	Blog/Webpage	UK	Maggie’s—Everyone’s home of cancer care (Cancer support organisation)
1	Your emotions after treatment—Dana Farber Cancer Institute https://www.dana-farber.org/for-patients-and-families/for-survivors/caring-for-yourself-after-cancer/your-emotions-after-treatment/ (accessed on 25 February 2020)	2020	Fair (64%)	Adequate (55.3%)	10	Webpage	USA	Dana Farber Cancer Institute (Medical/research center)
1	Cancer survivors: Managing your emotions after cancer treatment https://www.mayoclinic.org/diseases-conditions/cancer/in-depth/cancer-survivor/art-20047129 (accessed on 25 February 2020)	2018	Fair (61.3%)	Superior (76.3%)	10	Blog/Webpage	USA	Mayo Clinic Health System (Medical/research center)
1	6 tips for managing fear of cancer recurrence https://www.mskcc.org/news/six-tips-managing-fear-recurrence (accessed on 25 February 2020)	2014	Fair (61.3%)	Adequate (69%)	12	Blog/Webpage	USA	Memorial Sloan Kettering Cancer Centre (Medical/research center)
1	Life after treatment—fear of the cancer coming back https://www.cancervic.org.au/living-with-cancer/life-after-treatment/fear-of-the-cancer-coming-back (accessed on 25 February 2020)	2020	Fair (60%)	Adequate (64%)	10	PDF and Webpage	Australia	Cancer Council Victoria (Cancer support organisation)
1	Life after Cancer https://www.cancer.org/treatment/survivorship-during-and-after-treatment/be-healthy-after-treatment/life-after-cancer.html (accessed on 25 February 2020)	2016	Fair (57.3%)	Adequate (57.1%)	9	Webpage	USA	American Cancer Society (Cancer support organisation)
1	How to stop worrying about cancer returning https://www.netdoctor.co.uk/healthy-living/a28612/fear-of-cancer-recurrence/ (accessed on 25 February 2020)	2017	Fair (56%)	Adequate (64.3%)	11	Blog/Webpage	UK	Hearst UK National Magazine Company (Health education/publishing)
1	Is my cancer coming back?—How to cope with the FCR https://www.foxchase.org/blog/2018-03-23-how-to-cope-with-the-fear-of-a-cancer-recurrence (accessed on 25 February 2020)	2018	Fair (56%)	Adequate (63.2%)	9	Blog/Webpage	USA	Fox Chase Cancer Centre (Medical/research center)
1	FCR—Fact sheet https://www.bcna.org.au/media/4167/bcna-fact-sheet-fear-of-cancer-recurrence-jan-2017.pdf (accessed on 25 February 2020)	2017	Fair (54.7%)	Adequate (61.9%)	13	PDF/e-book	Australia	Breast Cancer Network Australia (Cancer support organisation)
1	Coping with fear of cancer recurrence https://www.cancercare.org/publications/253-coping_with_the_fear_of_recurrence# (accessed on 25 February 2020)	2019	Fair (53%)	Adequate (52.4%)	13	PDF and Webpage	USA	CancerCare (Cancer support organisation)
1	Fear of cancer recurrence https://www.bcna.org.au/understanding-breast-cancer/fear-of-cancer-recurrence/ (accessed on 25 February 2020)	2020	Poor (51%)	Adequate (60.5%)	11	Webpage	Australia	Breast Cancer Network Australia (Cancer support organisation)
1	Manage fear of cancer recurrence https://www.cancer.net/survivorship/life-after-cancer/coping-with-fear-recurrence (accessed on 25 February 2020)	2020	Poor (51%)	Adequate (59.4%)	11	Webpage	USA	American Society of Clinical Oncology (Professional association)
1	Coping with cancer—A new normal https://www.cancer.gov/about-cancer/coping/survivorship/new-normal (accessed on 25 February 2020)	2019	Poor (49.3%)	Adequate (61.9%)	9	Webpage	USA	National Cancer Institute (Medical/research center)
2	Understanding and managing the fear of cancer recurrence https://virginiacancer.com/cancer-survivorship/mental-health/understanding-and-managing-the-fear-of-cancer-recurrence/ (accessed on 25 February 2020)	2020	Fair (53%)	Adequate (45.2%)	13	Blog/Webpage	USA	Virginia Oncology Associates (Medical/research center)
2	6 tips to fight the fear of cancer returning https://www.eehealth.org/blog/2016/08/6-tips-to-fight-the-fear-of-cancer-returning/ (accessed on 25 February 2020)	2016	Poor (49.3%)	Adequate (47.6%)	10	Blog/Webpage	USA	Edward Elmhurst Health (Health education/publishing)
2	Treating fear of cancer recurrence https://www.cancertodaymag.org/Pages/cancer-talk/Treating-Fear-of-Recurrence.aspx (accessed on 25 February 2020)	2019	Poor (46.7%)	Adequate (40.5%)	13	Blog/Webpage	USA	American Association of Cancer Research (Professional association)
2	FCR—Mind, body tools offer help https://www.health.harvard.edu/blog/fear-of-cancer-recurrence-mind–body-tools-offer-hope-2019030716152 (accessed on 25 February 2020)	2019	Poor (45.3%)	Adequate (52%)	13	Blog/Webpage	USA	Harvard Health Publishing (Health education/publishing)
2	How to manage fear of cancer recurrence https://www.cancersupportcommunity.org/blog/2018/02/how-manage-fear-cancer-recurrence (accessed on 25 February 2020)	2020	Poor (45%)	Adequate (50%)	13	Blog/Webpage	USA	Cancer Support Community (Cancer support organisation)
2	Overcoming the anxiety as a cancer survivor https://www.henryford.com/blog/2017/06/overcoming-anxiety-cancer-survivor (accessed on 25 February 2020)	2017	Poor (38.7%)	Adequate (50%)	10	Blog/Webpage	USA	Henry Ford Live-Well (Health education/publishing)
2	Living in fear: Cancer recurrence https://www.mayoclinichealthsystem.org/hometown-health/speaking-of-health/living-in-fear-cancer-recurrence (accessed on 25 February 2020)	2019	Very Poor (30.7%)	Unsuitable (31%)	10	Webpage	USA	Mayo Clinic Health System (Medical/research center)
2	FCR is common, but oncologists can help https://www.curetoday.com/articles/fear-of-cancer-recurrence-is-common-but-oncologists-can-help (accessed on 25 February 2020)	2018	Very Poor (26.7%)	Unsuitable (31.6%)	14	Blog/Webpage	USA	Cure Today (Cancer support organisation)

* Resources were categorised into two clusters. Cluster 1: Higher quality and suitability; Cluster 2: Lower quality and suitability. DISCERN (quality): Superior = 70–100%, Adequate = 40–69%, Unsuitable < 39%, SAM (suitability): Excellent = 84–100%, Good = 68–83%, Fair = 52–67%, Poor = 36–51%, and Very poor < 35%.

**Table 3:** Mean Suitability Assessment of Materials ratings for included resources (*n* = 23).

SAM Categories	Mean	SD
Section 1: Content		
(1a) Purpose is evident	1.7	0.5
(1b) Content about behaviours	1.7	0.5
(1c) Scope is limited	1.7	0.5
(1d) Summary or review included	0.2	0.5
Section 1 total (max score 2)	1.3 (Adequate)	0.5
Section 2: Literacy Demand		
(2a) Reading grade level	0.2	0.4
(2b) Writing style	1.5	0.5
(2c) Common vocabulary	1.5	0.6
(2d) Context given first	1.8	0.4
(2e) Use of “road signs”	1.7	0.6
Section 2 total (max score 2)	1.3 (Adequate)	0.5
Section 3: Graphics		
(3a) Cover graphic shows purpose	0.7	0.5
(3b) Type of graphics	0.9	0.4
(3c) Relevance of illustrations	0.4	0.6
(3d) Lists, tables, etc. explained	1.4	0.6
(3e) Captions used for graphics	0	0
Section 3 total (max score 2)	0.7 (Not suitable)	0.4
Section 4: Layout and Typography		
(4a) Layout factors	1.3	0.6
(4b) Typography	1.8	0.4
(4c) Subheadings used	0.4	0.7
Section 4 total (max score 2)	1.2 (Adequate)	0.6
Section 5: Learning, stimulation, and motivation		
(5a) Interaction used	0.5	0.5
(5b) Behaviours modelled/specific	1.3	0.5
(5c) Motivation and self-efficacy	1.1	0.6
Section 5 total (max score 2)	1.0 (Adequate)	0.6
Section 6: Cultural Appropriateness		
(6a) Match in logic, language, and experience	1.0	1.0
(6b) Cultural images and examples *	NA	NA
Section 6 total (max score 2)	1.0 (Adequate)	1.0
SAM Total (range 0–42)	22.2	4.6
SAM Total (%)	56% (Adequate)	11.4

SAM: Suitability Assessment of Materials; SD: Standard Deviation; Category scoring: 2 = superior, 1 = adequate, 0 = not suitable; SAM score (%): 70–100% = “superior”, 40–69% = “adequate”, and <40% = “not suitable”. * Given none of the included resources targeted a specific cultural group, this criteria was deemed as not applicable (NA).

**Table 4:** Mean DISCERN ratings for included resources (*n* = 23).

DISCERN Categories	Mean (Range 1–5)	SD
**Reliability**		
Q1. Are the aims clear?	4.3	1.1
Q2. Does it achieve its aims?	3.9	1.0
Q3. Is it relevant?	4.0	0.8
Q4. Are the sources of information clear?	1.7	1.5
Q5. Is it clear when the information was produced?	2.0	0.7
Q6. Is it balanced and unbiased?	2.9	0.8
Q7. Are there details of additional sources?	2.6	1.3
Q8. Does it refer to areas of uncertainty?	2.0	0.9
**Treatment choices**		
Q9. Does it describe how the treatment works?	2.5	1.0
Q10. Does it describe the benefits of treatments?	2.3	0.9
Q11. Does it describe the risks of treatments?	1.0	0.2
Q12. Does it describe what would happen without treatment?	1.2	0.4
Q13. Does it describe how treatments affect quality of life?	2.8	1.1
Q14. Is it clear that there may be more than one treatment choice?	3.5	1.0
Q15. Does it support shared decision making?	3.0	0.8
Q16. **Overall quality rating**	2.9	1.1
**DISCERN score**		
DISCERN Total (range 15–75)	40	8.8
DISCERN Total (%)	53%	11.7

Item scoring: 1 to 5; Mean: Mean of 23 resources; SD: standard deviation. The bold headings in this table help differentiate the different measure sub scales.

## Data Availability

The data presented in this study are available on request from the corresponding author.

## Appendix A. Resource Characteristics

**Title**	**Resource Sponsor**	**Year Updated**	**Audio-Visual Elements**	**Advertisements**	**Self-Management Skills**
Fear of cancer recurrence	Not-for-profit organisation	2020	Yes	No	Yes
How to manage fear of cancer recurrence	Not-for-profit organisation	2020	No	No	Yes
Understanding and managing the fear of cancer recurrence	Health Organisation	2020	No	No	Yes
Life after treatment—fear of the cancer coming back	Not-for-profit organisation	2020	No	No	Yes
Coping with fear of cancer recurrence	Not-for-profit organisation	2019	No	No	Yes
Manage fear of cancer recurrence	Health Organisation	2020	Yes	No	Yes
Coping with the fear of cancer coming back (fear of cancer recurrence)	Health Organisation	2017	No	No	Yes
How to deal with FCR—patient treatment and support	Health Organisation	2011	No	No	Yes
FCR—Fact sheet	Not-for-profit organisation	2017	No	No	Yes
Coping with cancer—A new normal	Government funded	2019	No	No	Yes
How to stop worrying about cancer returning	Health Organisation	2017	No	Yes	NA
6 tips for managing fear of cancer recurrence	Health Organisation	2014	No	No	Yes
FCR—Mind, body tools offer help	University funded	2019	No	Yes	Yes
Treating fear of cancer recurrence	Not-for-profit organisation	2019	No	Yes	Yes
Overcoming the anxiety as a cancer survivor	Not-for-profit organisation	2017	No	Yes	Yes
6 tips to fight the fear of cancer returning	Not-for-profit organisation	2016	No	Yes	Yes
Living in fear: Cancer recurrence	Not-for-profit organisation	2019	No	No	Yes
Life after Cancer	Not-for-profit organisation	2016	No	No	Yes
FCR is common, but oncologists can help	Not-for-profit organisation	2018	No	Yes	Yes
Cancer survivors: Managing your emotions after cancer treatment	Not-for-profit organisation	2018	No	Yes	Yes
Is my cancer coming back?—How to cope with the FCR	Health Organisation	2018	No	Yes	Yes
Your emotions after treatment	Not-for-profit organisation	2020	No	Yes	Yes
Fear of cancer returning	Not-for-profit organisation	2020	No	No	Yes
